# Supplementary material for: Vast Gene Flow among the Spanish Populations of the Pest Bactrocera oleae (Diptera, Tephritidae), Phylogeography of a Metapopulation to Be Controlled and Its Mediterranean Genetic Context
Source: Insects. 2022 Jul 17;13(7):642. doi: 10.3390/insects13070642 (PMC9322173; doi:10.3390/insects13070642)
Supplement: Supplementary file 1 [file insects-13-00642-s001.zip › insects-1811810-supplementary.pdf]

	MAL	MAD	NAV	SAL1	TAR	BAD	BAL	CAC	COR	GER	GRA	JAE	SAL2	TER	VAL	POR1	POR2	ITA	TUN1	TUN2	TUN3	GRE1	GRE2	GRE3	ISR1	ISR2
**MAD**	0,000																									
**NAV**	0,006	0,000																								
**SAL1**	0,004	0,026	0,072																							
**TAR**	0,000	0,000	0,028	0,030																						
**BAD**	0,010	0,027	0,063	0,006	0,005																					
**BAL**	0,021	0,000	0,000	0,101	0,037	0,096																				
**CAC**	0,000	0,000	0,000	0,012	0,000	0,004	0,000																			
**COR**	0,000	0,002	0,004	0,019	0,000	0,036	0,034	0,000																		
**GER**	0,019	0,028	0,000	0,063	0,050	0,039	0,061	0,000	0,005																	
**GRA**	0,018	0,000	0,046	0,074	0,000	0,045	0,000	0,000	0,060	0,091																
**JAE**	0,000	0,000	0,023	0,035	0,000	0,015	0,042	0,000	0,000	0,051	0,000															
**SAL2**	0,016	0,000	0,000	0,071	0,010	0,066	0,000	0,000	0,000	0,006	0,032	0,001														
**TER**	0,009	0,019	0,000	0,048	0,046	0,060	0,042	0,000	0,000	0,000	0,100	0,048	0,000													
**VAL**	0,000	0,000	0,000	0,018	0,000	0,015	0,051	0,000	0,000	0,000	0,063	0,006	0,006	0,000												
**POR1**	0,000	0,000	0,000	0,031	0,000	0,031	0,000	0,000	0,000	0,010	0,000	0,000	0,000	0,017	0,000											
**POR2**	0,013	0,022	0,000	0,047	0,050	0,041	0,079	0,011	0,000	0,000	0,116	0,051	0,018	0,000	0,000	0,044										
**ITA**	**0,345**	**0,339**	0,220	**0,338**	**0,386**	0,258	**0,381**	**0,268**	**0,290**	0,107	**0,366**	**0,329**	0,245	0,215	0,186	**0,322**	0,146									
**TUN1**	**0,337**	**0,320**	**0,232**	**0,337**	**0,366**	**0,276**	**0,350**	**0,261**	**0,297**	0,158	**0,345**	**0,325**	**0,250**	**0,243**	0,199	**0,312**	0,178	0,039								
**TUN2**	**0,257**	0,241	0,185	**0,260**	0,280	**0,222**	**0,284**	0,199	0,227	0,136	**0,284**	**0,253**	**0,202**	0,190	0,133	0,249	0,126	0,121	0,000							
**TUN3**	0,135	0,116	0,000	**0,181**	0,176	0,144	0,122	0,059	0,088	0,000	0,178	**0,145**	0,029	0,016	0,015	0,092	0,014	0,127	**0,158**	0,148						
**GRE1**	**0,390**	**0,375**	**0,307**	**0,383**	**0,417**	**0,328**	**0,412**	**0,321**	**0,352**	**0,237**	**0,398**	**0,377**	**0,315**	**0,313**	0,256	**0,372**	0,243	0,126	0,000	0,000	**0,250**					
**GRE2**	**0,282**	**0,274**	0,185	**0,282**	**0,318**	**0,230**	**0,319**	**0,209**	0,236	0,107	**0,315**	**0,280**	**0,212**	0,190	0,136	**0,274**	0,115	0,036	0,000	0,000	0,114	0,000				
**GRE3**	**0,717**	**0,700**	**0,696**	**0,697**	**0,745**	**0,646**	**0,729**	**0,663**	**0,675**	**0,643**	**0,695**	**0,692**	**0,650**	**0,671**	**0,625**	**0,689**	**0,603**	**0,650**	**0,424**	0,278	**0,711**	0,338	**0,435**			
**ISR1**	**0,735**	**0,719**	**0,718**	**0,716**	**0,754**	**0,672**	**0,743**	**0,689**	**0,696**	**0,675**	**0,712**	**0,709**	**0,676**	**0,695**	**0,659**	**0,710**	**0,640**	0,684	**0,477**	0,318	**0,732**	0,407	**0,504**	0,093		
**ISR2**	**0,783**	**0,766**	**0,768**	**0,760**	**0,807**	**0,713**	**0,791**	**0,735**	**0,741**	**0,720**	**0,756**	**0,756**	**0,719**	**0,741**	**0,704**	**0,754**	**0,681**	**0,736**	**0,525**	**0,359**	**0,789**	**0,455**	**0,556**	0,075	0,000	
**ISR3**	**0,732**	**0,716**	**0,716**	**0,714**	**0,752**	**0,671**	**0,740**	**0,687**	**0,696**	**0,673**	**0,711**	**0,709**	**0,675**	**0,694**	**0,658**	**0,708**	**0,641**	**0,682**	**0,473**	0,307	**0,729**	**0,410**	**0,509**	**0,169**	0,000	0,002

Table S1. F_{ST} fixation indices among the *olf* populations analysed. Statistically significant values ($p < 0.05$) after applying Bonferroni corrections are shown in bold.

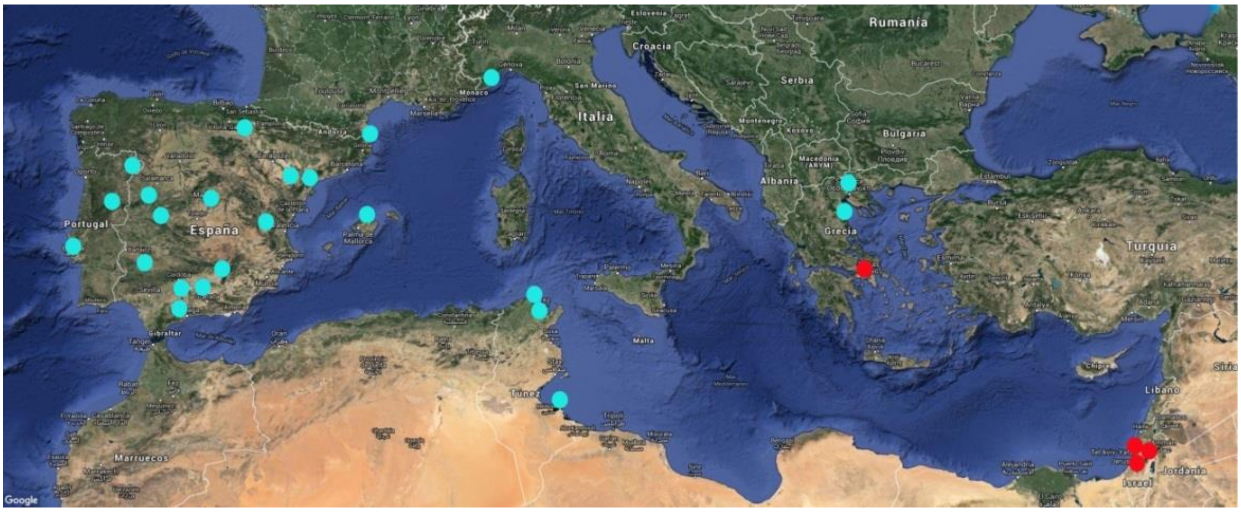

Figure S1. SAMOVA analysis with the most probable genetic groups for the *B. oleae* populations sampled.

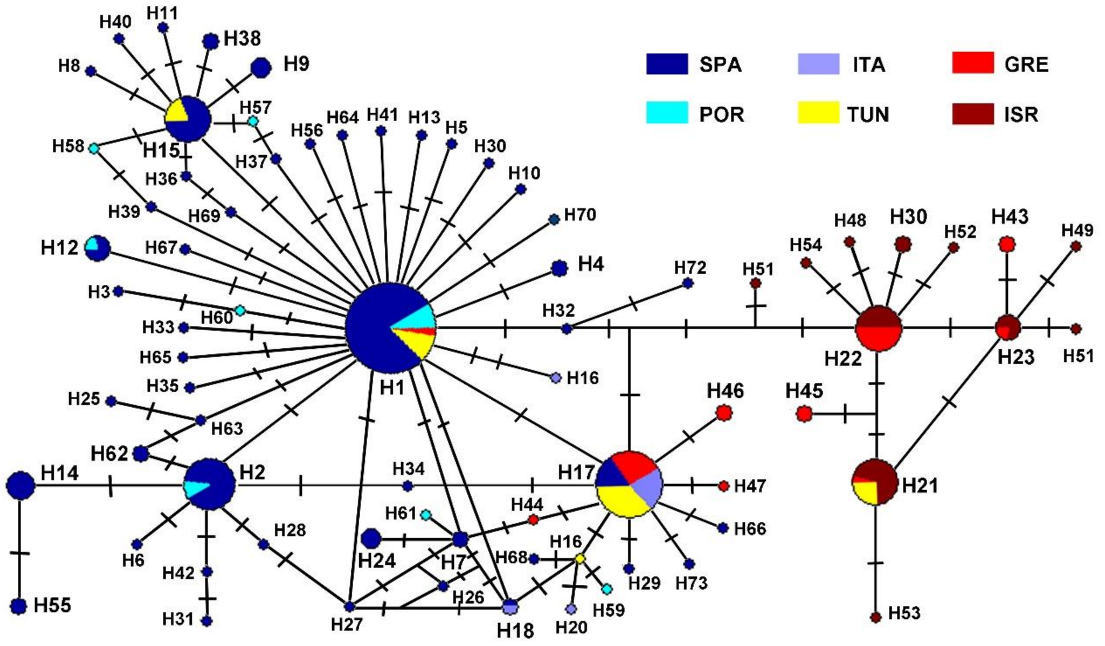

Figure S2. Network of the 73 haplotypes detected in the *B. oleae* populations analysed. Countries are identified by colour. Bars indicate the mutational steps separating the different haplotypes. Intersections represent unsampled haplotypes. H17 is the designated ancestral haplotype.

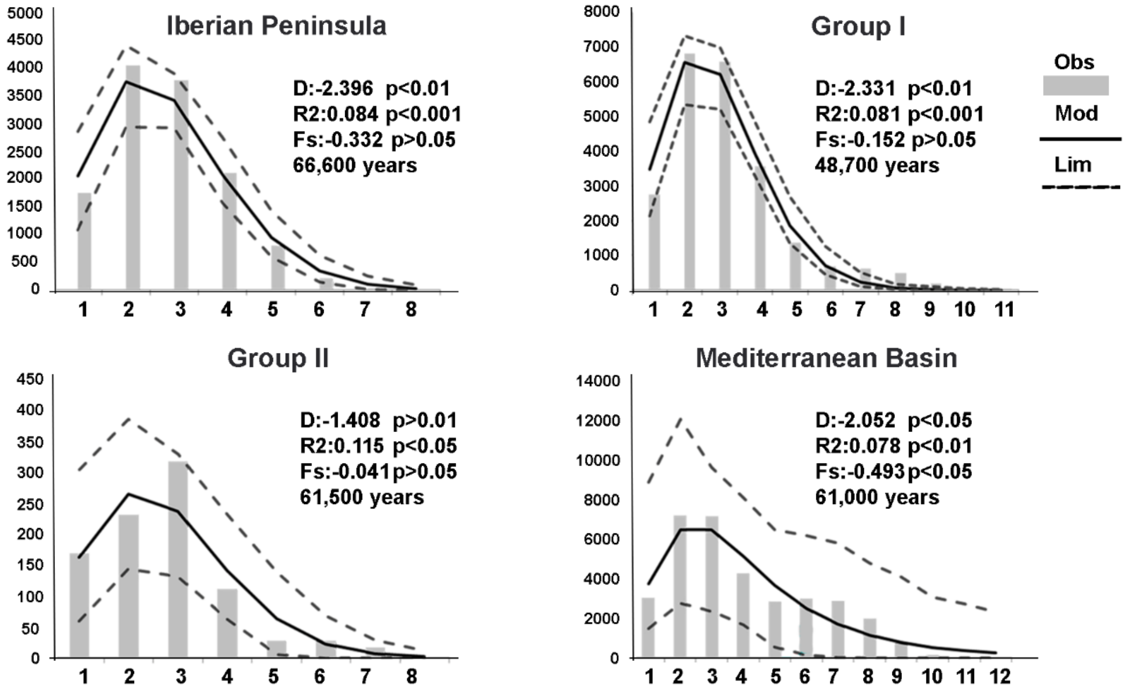

Figure S3. Mismatch distributions, observed and expected, in the Spanish populations as a whole and in the ‘Western and ‘Eastern genetic clusters detected. The neutrality test Fs, D R2 values and time elapsed since last expansion are also shown
